# Supplementary material for: Milk-Derived miR-22-3p Promotes Proliferation of Human Intestinal Epithelial Cells (HIECs) by Regulating Gene Expression
Source: Nutrients. 2022 Nov 19;14(22):4901. doi: 10.3390/nu14224901 (PMC9695551; doi:10.3390/nu14224901)
Supplement: Supplementary file 1 [file nutrients-2015180-supplementary.pdf › nutrients-2015180-supplementary.pdf]

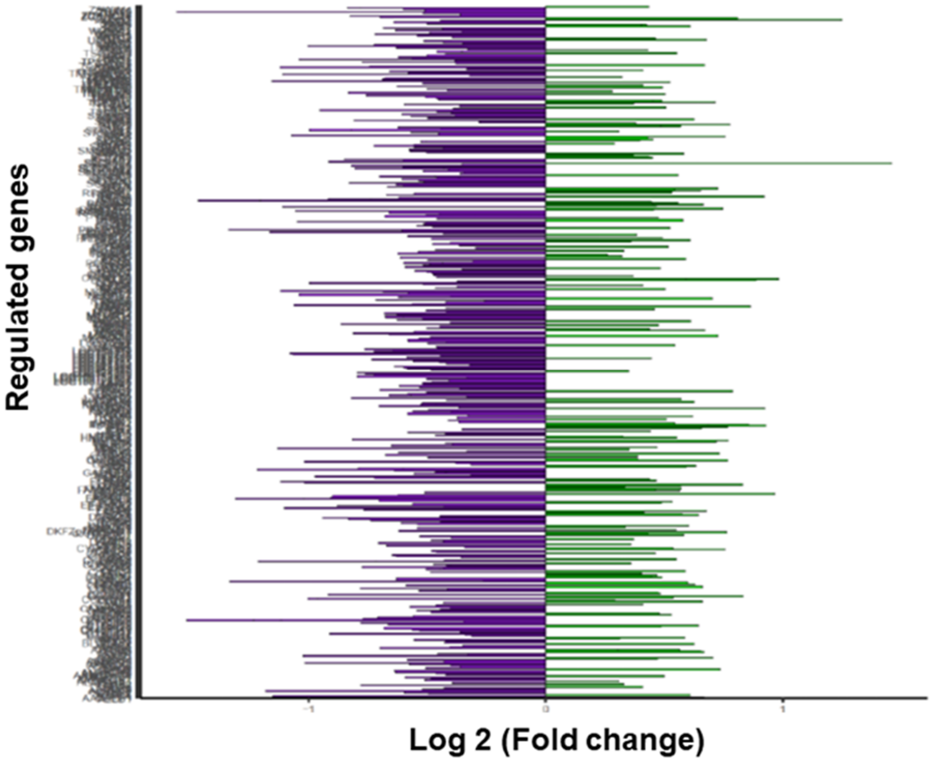

Supplementary Figure S1. miR-22 significantly influences gene transcription in HIECs. Totally, 608 genes were regulated. Green lines represent up-regulated genes (190), and purple lines stand for down-regulated genes (418). $p<0.05$.
